# Early Echocardiographic and Cardiac MRI Findings in Multisystem Inflammatory Syndrome in Children

**DOI:** 10.3390/jcm10153360

**Published:** 2021-07-29

**Authors:** Domenico Sirico, Alessia Basso, Elena Reffo, Annachiara Cavaliere, Biagio Castaldi, Jolanda Sabatino, Alessandra Meneghel, Giorgia Martini, Liviana Da Dalt, Francesco Zulian, Giovanni Di Salvo

**Affiliations:** 1 Pediatric and Congenital Cardiology Unit, Department for Women’s and Children’s Health, University Hospital of Padova, 35128 Padua, Italy; alessia.basso126@gmail.com (A.B.); elena.reffo@aopd.veneto.it (E.R.); biagio.castaldi@aopd.veneto.it (B.C.); jolanda.sabatino@unipd.it (J.S.); giovanni.disalvo@unipd.it (G.D.S.); 2 Institute of Radiology, University Hospital of Padova, 35128 Padua, Italy; annachiara.cavaliere@aopd.veneto.it; 3 Pediatric Rheumatology Unit, Department for Women’s and Children’s Health, University Hospital of Padova, 35128 Padua, Italy; alessandra.meneghel@aopd.veneto.it (A.M.); giorgia.martini@aopd.veneto.it (G.M.); francesco.zulian@unipd.it (F.Z.); 4 Pediatric Emergency Unit, Department for Women’s and Children’s Health, University Hospital of Padova, 35128 Padua, Italy; liviana.dadalt@unipd.it

**Keywords:** multisystem inflammatory syndrome in children, COVID 19, speckle tracking echocardiography, longitudinal strain, cardiac magnetic resonance, myocardial injury

## Abstract

Multisystem Inflammatory Syndrome in Children (MIS-C) is a known severe condition affecting children previously exposed to SARS-CoV-2. The aim of our study was to describe the early cardiac abnormalities in patients with MIS-C, evaluated by speckle tracking echocardiography (STE) and cardiac MRI (CMR). Clinical, laboratory and microbiological data were measured for all patients. All children underwent standard transthoracic echocardiography, STE with analysis of left ventricle global longitudinal strain (GLS). Seventeen (75%) of the children were evaluated with CMR. Twenty-three patients (13M, 10F) were recruited, mean age was 8.1 ± 4 years. Cardiovascular symptoms were present in 10 (43.5%). Nine children (39.1%) shared Kawasaki Disease-like symptoms. Four patients (17.4%) needed ICU admission. In-hospital survival was 100%. TnI was elevated in 15 (65.2%) and BNP in 20 (86.9%) patients. The median time to STE evaluation was 8 days and to CMR was 18 days after fever onset. Mean LVEF was 59 ± 10%. Coronary dilation was observed in six (26.1%) patients. STE showed a reduced mean LVGLS (−17 ± 4.3%). LGE with a non-ischemic pattern was evident in six out of seventeen patients (35.2%). The elevation of myocardial necrosis markers, the reduction of LVGLS and the presence of LGE on CMR in about a quarter of MIS-C patients supports the hypothesis of a post-viral immune-mediated myocarditis-like pathogenesis.

## 1. Introduction

Since the beginning of 2020, the coronavirus disease 2019 (COVID-19) pandemic, caused by the severe acute respiratory syndrome coronavirus 2 (SARS-CoV-2) infection, has represented the greatest health care concern worldwide. Although initial reports showed less severe COVID-19 acute manifestations among children compared to adults [1,2,3,4], growing evidence has been produced regarding a severe systemic hyperinflammation syndrome, named Multisystem Inflammatory Syndrome in Children (MIS-C), affecting children and adolescents exposed to SARS-CoV2 from 2 to 6 weeks earlier [5]. The characteristic features of the disease are fever, gastrointestinal symptoms, muco-cutaneous inflammation signs and myocardial and coronary artery involvement. Interestingly, this multisystemic illness shares features with other pediatric inflammatory conditions, such as Kawasaki disease (KD), bacterial sepsis, toxic shock syndrome and macrophage activation syndrome [6]. However, hemodynamic shock, myocardial dysfunction and gastrointestinal involvement are peculiar findings of this unique syndrome [7]. Cardiovascular manifestations in MIS-C are common, occurring in 34–82% of cases [8]. They include myocardial dysfunction, coronary artery dilation or aneurysms, arrhythmia, conduction abnormalities, pericarditis and valvulitis. Severe cases can present with distributive or cardiogenic shock requiring fluid resuscitation and inotropic support [9,10]. Among the others, left ventricular (LV) systolic dysfunction is the most frequent cardiac abnormal finding. Latest evidence shows that myocardial dysfunction improves early after disease onset and that myocardial injury may not be appreciated on cardiac MRI just a few weeks after symptoms onset, suggesting an immune-mediated pathogenesis of such condition [11,12]. 

The aim of our study was to assess cardiac involvement in a cohort of MIS-C patients by two advanced cardiovascular imaging techniques, speckle tracking echocardiography and cardiac MRI, early after disease onset.

## 2. Materials and Methods

### 2.1. Study Design and Population

This is a retrospective observational, single-center study performed at the Department for Women’s and Children’s Health (W&CHD) of Padua University Hospital, Italy. We evaluated patients who were admitted to our hospital with the diagnosis of MIS-C from the 23rd of April 2020 to the 25th of February 2021. The diagnosis of MIS-C was made according to the WHO case definition [5]. 

All subjects were tested for SARS-CoV-2 infection by RT-PCR on nasopharyngeal swab on admission. Serological assay included IgG and IgM detection, targeting a recombinant nucleocapsid (N)-spike (S) protein of SARS-CoV-2 (IgM cut-off 1.0 AU/mL, IgG cut-off 1.1 AU/mL). 

All patients underwent routine blood tests including blood cell counts, biochemical profile and markers of systemic inflammation (C-reactive protein-CRP, erythrocyte sedimentation rate (ESR), procalcitonin, ferritin, D-dimer, fibrinogen, LDH). Troponin I (TnI, normal value <34 ng/L) and Brain Natriuretic Peptide (BNP, normal value <100 ng/L) were measured on admission and serially during hospital stay.

The admission into the Intensive Care Unit (ICU) was based on clinical and hemodynamic status at presentation. Pharmacological treatment was established following the Clinical Guidance of the American College of Rheumatology for Pediatric Patients with MIS-C [13]. All patients received intravenous immunoglobulins (IVIGs), intravenous corticosteroids (methylprednisolone) and antiplatelet therapy (aspirin). The use of a biologic agent (Anakinra) was reserved for patients with severe or critical illness refractory to the standard therapy. One patient with giant coronary artery aneurysms and thrombosis required thrombolysis with t-PA, anticoagulant therapy and adjunctive antiplatelet drug.

### 2.2. Echocardiography

All children underwent full cardiac evaluation, including ECG and transthoracic echocardiogram (TTE), during the first days of hospitalization. Two-dimensional standard echocardiography was performed by experienced cardiac sonographers using the GE Vivid E9 Ultrasound System (GE Healthcare, Chicago, IL) following the recommendations for cardiovascular imaging during COVID- 19 pandemic [14,15].

Standard echocardiographic parameters include left ventricle ejection fraction (LVEF) using Simpson’s biplane method, the early and late mitral inflow peak velocities by spectral Doppler, right ventricular (RV) function using Fractional Area Change (FAC), tricuspid annular plane systolic excursion (TAPSE) by M-mode and the early diastolic septal and lateral mitral annular peak velocities and lateral tricuspid annular peak velocity by TDI. Furthermore, coronary arteries were assessed following the AHA Guidelines for Kawasaki Disease [16]. Coronary artery abnormalities were classified using the Boston z-score system as follows: no involvement <2, dilation ≥2 to <2.5, small aneurysm ≥2.5 to <5, medium aneurysm ≥5 to <10, large or giant aneurysm ≥10. 

Longitudinal strain (LS) analysis of the left ventricle, through 2D STE analysis, was performed offline using GE EchoPac Software (GE Healthcare, Chicago, IL), as previously described [17]. Briefly, the best apical four-, two- and three-chamber views to visualize the left ventricle segments were selected. Afterwards, 3 points (2 annular and 1 apical) were positioned, enabling the software to track the myocardium semi-automatically throughout the heart cycle. The region of interest was adjusted with careful inspection of the endocardial border, and manual correction was performed if needed. The automated algorithm allowed global longitudinal strain (GLS) to be calculated. Left ventricle LS by speckle tracking was defined as the average peak negative value on the strain curve during the systole (end of T wave on the ECG) of all the studied segments [18]. The peak negative systolic strain value for each regional LV segment was also analyzed.

Analysis of the standard TTE and STE was performed by two experienced echocardiographers blind to the clinical data. The reproducibility data of our Echo Lab for standard TTE parameters as well as for STE has been published already [19].

### 2.3. Cardiac MRI

Seventeen (74%) patients underwent CMR. The scan was performed with a 1.5-Tesla scanner (Philips Achieva, Philips Healthcare, Best, Netherlands). Sedation with intravenous midazolam or ketamine was required for patients younger than 6 years old. CMR included cine SSFP and T2-weighted (T2W) images with fat-suppression. First pass perfusion with intravenous administration of contrast agent (0.1 mmol/kg body weight gadoterate meglumine) was realized in all patients. Late gadolinium-enhanced (LGE) sequences, in short axis and four-chambers planes, were acquired 10 minutes after contrast administration using a standard 2-dimensional breath-hold phase-sensitive inversion recovery sequence with the inversion time selected to null the myocardial signal.

In all patients, LV and RV end-diastolic volume, end-systolic volume and ejection fraction were measured. The CMR images were analyzed for detecting the presence of myocardial edema and fibrosis using standard LV 17-segment model and specifying the pattern (transmural, subendocardial, subepicardial).

To rule out coronary dilatation or aneurysm, 3D SSFP isovolumetric MRA ECG and navigator gating was performed. Other findings, such as pericardial or pleural effusion and the presence of coronary thrombosis, were reported.

### 2.4. Statistical Analysis

Categorical variables were presented as percentage (%), and continuous variables as mean ± standard deviation. Shapiro–Wilk test and histogram were used to test normality for each variable. Student’s *t*-test was performed for normally distributed continuous variables and Mann–Whitney *U* test for nonparametric continuous variables. Chi-square test or Fisher’s exact test were performed for categorical variables to examine if there were significant differences between the groups. Statistical analysis was performed using SPSS 21.0 (IBM, Armonk, NY, USA).

## 3. Results

Twenty-three children (10 females, 13 males) with confirmed MIS-C diagnosis entered the study (Table 1). Mean age at diagnosis was 8.1 ± 4 years, mean BSA 1.06 ± 0.35 m^2^. The majority was of Caucasian origin (79%), the remaining had African (17%) or Asian (4%) ethnicity. Eighteen patients (78%) had serological-confirmed SARS-CoV-2 infection, and most of them had no documented underlying conditions. Nine children (39%) shared Kawasaki Disease (KD)-like symptoms. The multisystem involvement, treatment and laboratory findings are summarized in (Table 2). In four cases (17%) admission in ICU was needed and three of these required inotropic support. Regarding cardiovascular symptoms, seven patients (30.4%) presented with hypotension, and seven (30.4%) with sinus bradycardia. In-hospital survival was 100%. All patients showed a hyperinflammatory state characterized by elevated CRP, ESR and D-Dimer. TnI was abnormal (>34 ng/L) in 15 children (65.2%). BNP was significantly elevated in 20 (87%) patients (mean value 581 ± 736 pg/mL). 

ECG was reported as abnormal in 52% of cases, of which eight patients with abnormalities of the repolarization phase: negative T waves in six patients, mostly in the lateral and inferior leads, and ST segment depression in two patients in the lateral leads. Seven patients (30.4%) presented with sinus bradycardia, while two (8.7%) showed atrioventricular block (1st degree block in one and 2nd degree Mobitz in the other).

**Figure 1:**
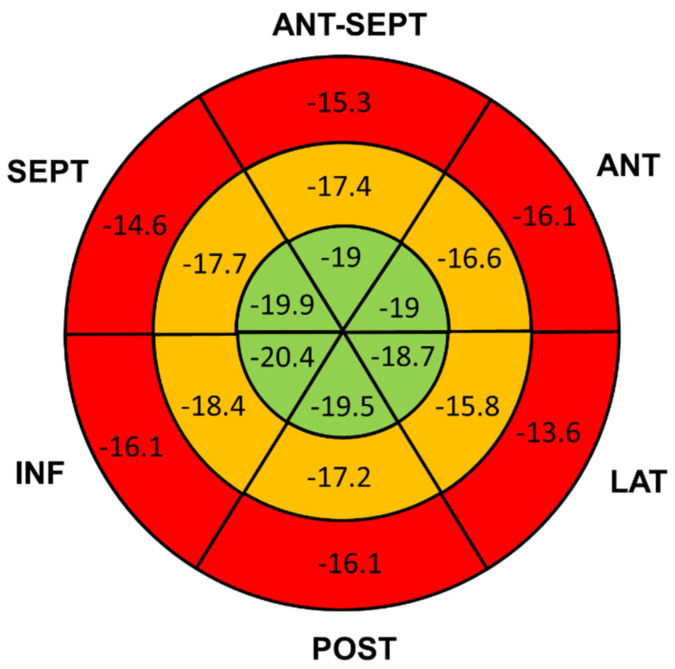
Left ventricle GLS Bull’s Eye. Bull’s Eye representation of mean longitudinal strain values (%) for each segment of the left ventricle. Basal segments (red) showed the most impaired deformation compared to mid-wall (orange) and apical (green) segments. GLS (global longitudinal strain).

All patients underwent echocardiographic evaluation with a median time of 8 days since fever onset and 1.5 days since hospital admission. Mean LVEF was 59 ± 10%, RV FAC 45 ± 7% and TAPSE 18.8 ± 4.7mm. Coronary dilation (z-score > 2) was detected in six patients (26.1%), of which five had mild to moderate dilation (2 < z-score < 4). One 4-year-old child showed severe left descending artery dilation (z-score +20) with giant aneurysm and coronary thrombosis leading to acute anterior and lateral myocardial infarction and acute heart failure necessitating thrombolysis with t-PA, anticoagulant therapy and adjunctive antiplatelet drug. Pericardial effusion was described in six patients (26%). STE showed reduced LVGLS −17 ± 4.3%, and 74% of patients presented at least two LV segments with a longitudinal strain higher than −16%. Basal and mid LV segments were the most affected with 53% and 41% having a longitudinal strain higher than −16%, respectively, as compared to the apical ones (18%) (Figure 1). 

Median time to CMR was 18 days from fever onset and 11 days from hospital admission. LVEF on CMR was 60 ± 13%, LGE with non-ischemic pattern was evident in six out of seventeen patients (35.3%) who underwent CMR, and all of them had the imaging performed within 19 days of the symptom’s onset. Pericardial effusion was observed in two patients (11.7%). Only the patient who experienced myocardial infarction showed edema on CMR.

**Figure 2:**
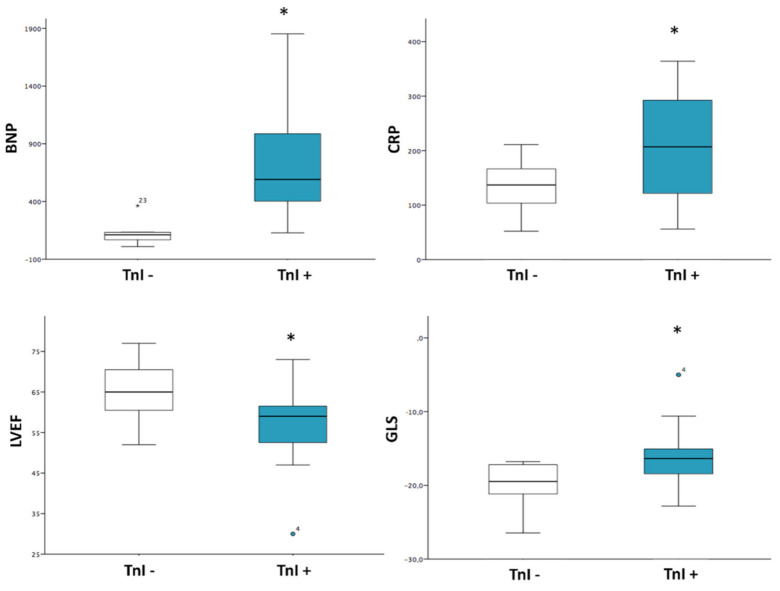
MIS-C and Myocardial Injury. Patients with elevated TnI levels (>34 ng/L) showed significantly higher levels of BNP and CRP, lower LVEF and less negative GLS values compared to patients with normal TnI levels. TnI (troponin I); BNP (brain natriuretic peptide); CRP (C-reactive protein); LVEF (left ventricle ejection fraction); GLS (global longitudinal strain). * = *p* < 0.05.

Patients with elevated TnI levels (>34 ng/L) showed significantly higher levels of circulating BNP compared to patients with normal TnI levels (824 ± 815 vs. 125 ± 105, *p*-value 0.03). The two groups did not show significant differences in SARS-CoV-2 IgG titles, nor in inflammatory markers (ESR, D-Dimer, Ferritine), except for CRP (205 ± 104 vs. 135 ± 49, *p* = 0.038). On echocardiography, patients with higher TnI levels displayed significantly lower LVEF (56 ± 10% vs. 65 ± 8%, *p*-value 0.042) and less negative LVGLS (−16.1 ± 4.2% vs. −19.8 ± 3.2%, *p*-value 0.042) values, while there was no difference regarding RV systolic functional parameters (TAPSE: 20 ± 4.9 mm vs. 16.4 ± 3.4 mm, *p*-value 0.09; RV FAC: 46 ± 8.2% vs. 44 ± 4.9%, *p*-value 0.63), or presence of coronary artery dilation (X^2^ = 0.52; *p* = 0.47). Finally, the two groups were similar regarding the evidence of LGE on CMR (X^2^ = 0.01; *p*= 0.91). (Table 3; Figure 2).

## 4. Discussion

MIS-C has been recently described in pediatric patients with previous SARS-CoV-2 infection, raising concerns for a population which was previously considered to be relatively spared from COVID-19 consequences. Usually, this systemic hyperinflammation illness develops 2–6 weeks after Coronavirus infection in about 0.6% of patients [20]. Interestingly, the severity of clinical manifestations is not related to the gravity of the previous viral disease. Moreover, in addition to fever, universally present, the syndrome shows multisystem involvement including cutaneous, abdominal and cardiovascular manifestations [21]. The latter can occur in up to 80% of patients and can range from minor involvement to cardiovascular collapse and shock. 

**Figure 3:**
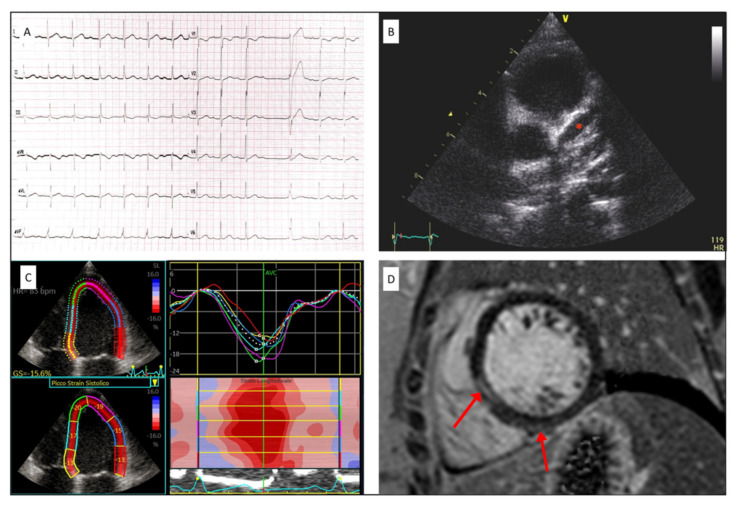
Cardiac findings in MIS-C patients. (**A**) First degree atrioventricular block with supraventricular ectopic beats; (**B**) giant aneurysm of the left anterior descending artery (red star); (**C**) strain curves in a multisystem inflammatory syndrome in children (MIS-C) patient during acute phase showing reduced global longitudinal strain (GLS); (**D**) intramural LGE enhancement in the septal and infero-lateral LV walls (red arrows).

To the best of our knowledge, this is the first report combining echocardiographic and CMR data during the early acute phase of MIS-C (Figure 3). In our cohort, myocardial injury and subsequent LV systolic dysfunction seems to be the most frequent early cardiac abnormalities [22]. Two thirds (65%) of our cohort presented myocardial injury diagnosed on the basis of troponin elevation, which was consistent with previous reports [23]. 

### 4.1. LV Longitudinal Strain 

The analysis of LV myocardial longitudinal deformation showed a decrease in GLS values in our population, which was more pronounced in patients with higher TnI levels. In agreement with Matsubara and colleagues, the reduction in longitudinal strain was present even in patients with preserved LVEF, suggesting subclinical myocardial dysfunction [11]. Interestingly, we found a base to apex gradient in LV regional LS abnormalities, with apical segments being less affected. This pattern has already been described in other cardiac diseases such as systemic hypertension [24], and recently reported by our group in a cohort of pediatric patients with previous asymptomatic or mildly symptomatic COVID-19 without MIS-C development [25]. Of interest, other studies on different populations have already demonstrated the prognostic value of GLS [26]. In this regard, our findings of impaired GLS in a substantial portion of our MIS-C cohort is of concern and may have implications for the restart of sporting activities. Thus, long-term follow up studies are needed.

### 4.2. Cardiac Magnetic Resonance 

Furthermore, early CMR showed delayed enhancement in a quarter of evaluated patients with nodular, subepicardial or mesocardial patterns involving, more frequently, the mid-wall (*n* = 14 segments) and, less frequently, the apical segments (*n* = 8 segments). LGE patterns displayed in our patients are typical for non-ischemic or myocarditis-like physiopathology. Conversely to what was reported in a recent small case series of MIS-C (*n* = 4) [27], we did not report the presence of myocardial edema on T2 sequences. A possible explanation might be the timing of CMR, which was carried out earlier (<11 days) in the case series as compared to our study. Consistently, a recent study describing CMR findings in 20 patients with MIS-C, reported normal T1 and T2 mean values and presence of LGE in only two (10%) patients [12]. Nevertheless, the mean time from fever onset to CMR was 27 ± 14 days, much longer than in our study. All together these CMR findings suggest that MIS-C may induce myocardial injury with myocarditis-like damage in the very early phase of the disease, with subsequent rapid improvement within a few weeks of symptoms onset, on appropriate immunomodulatory treatment.

### 4.3. MIS-C and Kawasaki Disease 

MIS-C is known to share some of the clinical, laboratory and instrumental features of Kawasaki’s disease (KD). However, when compared with the latter, MIS-C patients are usually older, present higher inflammatory markers levels, higher troponin elevation and lower incidence of coronary involvement [28]. Our population mean age was 8 years, higher than the usual age of presentation for KD in our country [29]. Myocardial injury (TnI +) was observed in 65% of our patients, significantly higher than the usual rate in KD patients (5%) [23]. In agreement with previous reports, 40% of our population presented with overlapping symptoms or features of KD. Interestingly, these patients did not differ significantly from the remaining patients in any of the anthropometric, laboratory or imaging variables, meaning that, even sharing some clinical features, the two conditions should probably be considered as different entities. However, a quarter of our population showed coronary artery dilation (z-score > 2), and one patient developed giant coronary artery aneurysms with thrombosis and subsequent extended myocardial infarction of the left ventricle [30]. Of note, this patient was diagnosed as having KD and treated with just IVIG in a peripheral hospital. This finding highlights the importance of an early and proper diagnosis and treatment of MIS-C, since coronary artery abnormalities (CAAs), reported in 9–24% in this contest, are mild and reversible in most patients [28,31,32].

### 4.4. Physiopathologic Hypothesis

**Figure 4:**
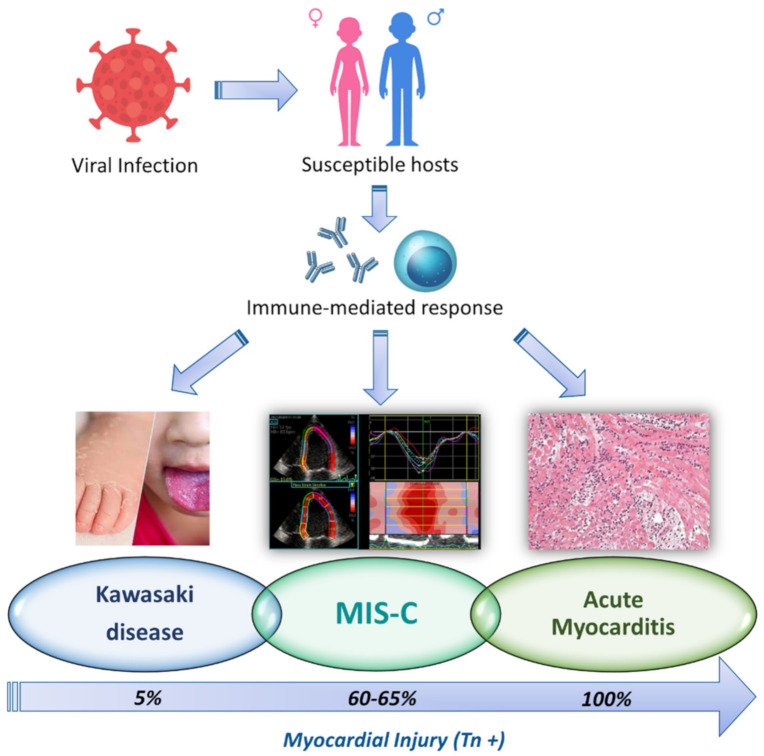
Physiopathological hypothesis of myocardial injury. In genetically susceptible individuals, viral infection may cause an abnormal immune-mediated response with subsequent myocardial injury. Kawasaki Disease (KD), MIS-C and acute post-viral myocarditis might share the same immune-mediated trigger to myocardial injury, although the incidence varies significantly among the different entities.

Although the cause of KD still remains unknown, approximately 9% of patients with KD have a recent history of respiratory infections [21]. Furthermore, seasonal variation, epidemiological clustering and a very low risk of recurrence suggest that infectious agents may be the main trigger of the KD immune response that is protective against future exposure in most patients [16,33]. There is evidence that an infection with a novel RNA virus through the upper respiratory tract may be responsible for the KD pathogenesis [34]. However, host genetic factors might be involved in the pathophysiology of KD, resulting in strong activation of the innate immune system [35]. Our study does not focus on the physiopathological mechanisms underlying MIS-C. However, the timing of the disease development in relation to SARS-CoV-2 infection, the presence of high levels of antibodies against the virus and the negativity of NPS in the majority of patients suggest the possibility that this disorder may be the result of an aberrant immunological response to a known agent in genetically susceptible hosts [21]. Interestingly, respiratory viruses, such as influenza and coronaviruses, are examples of pathogens that can trigger an immune-mediated lymphocytic myocarditis without evidence of a viral genome on endomyocardial biopsy [36]. Finally, the data regarding low mortality rate, rapid echocardiographic recovery and the absence of inflammation or LGE on CMR just a few weeks after disease onset, corroborate the hypothesis of an immune-mediated myocarditis-like damage in MIS-C population [11,12]. MIS-C may represent the missing physiopathologic link between KD and post-viral acute myocarditis, suggesting that virus-triggered immune-mediated reactions in susceptible hosts may represent the main cause of cardiomyocyte injury rather than actual direct virus-mediated cell injury, as previously hypothesized [37] (Figure 4).

### 4.5. Limitations

The single-center nature of our study may constitute a limitation. However, this increases the reproducibility and decreases the inter-observer variability of our measurements. Another possible limit may be that speckle tracking analysis was limited to the assessment of the longitudinal cardiac deformation. To this purpose, multiple studies have demonstrated the strong prognostic value of longitudinal deformation and, on the other side, the clinical value of circumferential and radial strain has yet to be proven. The lack of a control group may represent a limitation; however, this is secondary to the difficulty in enrolling age- and gender-matched pediatric patients, due to time constrictions and reduced activity in the outpatient clinic department secondary to COVID-19 pandemic.

## 5. Conclusions

STE and CMR were shown to be sensitive and specific tools to evaluate the early cardiac involvement in patients with MIS-C. The elevation of myocardial necrosis markers and myocardial injury, confirmed by reduced global and regional LV deformation together with the presence of LGE on CMR in about a quarter of the patients, support the pathogenetic hypothesis of a post-viral immune-mediated myocarditis. Although this syndrome shows an excellent short-term outcome if properly treated, the cardiac residual long-term damage needs to be determined.

## Figures and Tables

**Table 1:** Clinical characteristics of the patients. Values are numbers (%) or mean ± standard deviation. Legend: CHD (congenital heart disease), BNP (brain natriuretic peptide); CPR (C-reactive protein); ERS (Erythrocyte Sedimentation Rate); GI (gastrointestinal symptoms); Hb (hemoglobin); ICU (intensive care unit); IV (intravenous); IVIG (intravenous immunoglobulins); KD (Kawasaki disease); LDH (lactic dehydrogenase); PCT (procalcitonin); PLTs (platelet count); NP swab (nasopharyngeal swab); TnI (troponin I); WBC (white blood count).

	MIS-C Patients (*n* = 23)
**Age at onset (y** **ea** **rs)**	8.1 ± 4
**Male**	13 (56)
**Comorbidities**	2 (8.7)
Associated CHD	1 bicuspid aortic valve
**positive SARS-CoV-2 NP swab**	4 (17)
**positive SARS-CoV-2 IgG title**	18 (78)
**CLINICAL MANIFESTATIONS**	
Fever	23 (100)
GI	19 (82)
Muco-cutaneous	19 (82)
Neurological	6 (26)
Hypotension	7 (30)
Sinus bradycardia	7 (30)
KD like	9 (39)
ICU admission	4 (17)
**LABORATORY**	
TnI ≤ 34 ng/L	8 (35)
TnI > 34 ng/L	15 (65)
BNP (pg/mL)	581 ± 736
CRP (mg/L)	181 ± 94.4
PCT (ng/mL)	53 ± 102
ESR (mm/h)	57.9 ± 36.2
Ferritin (mcg/L)	882.8 ± 662.8
D-dimer (mcg/mL)	2311.6 ± 2853
LDH (U/L)	336.6 ± 93

**Table 2:** Early abnormal cardiac findings in MIS-C. Values are numbers (%) or mean ± standard deviation. Legend: AV block (atrioventricular block); GLS (global longitudinal strain); LVEF (left ventricular ejection fraction); LV LGE (left ventricle late gadolinium enhancement); MIS-C (multisystem inflammatory syndrome in children); RV FAC (right ventricle fractional area change); RVEF (right ventricular ejection fraction); TAPSE (tricuspid annular plane systolic excursion).

	MIS-C Patients (*n* = 23)
**EKG**	
EKG abnormalities	11 (48)
Ripolarization abnormalities	8 (35)
Sinus bradycardia	7 (30)
AV block	2 (9)
**ECHOCARDIOGRAPHY**	
LVEF (%)	59 ± 10
GLS (%)	−17 ± 4.3
E/A ratio	1.9 ± 0.5
Deceleration time (msec)	147 ± 31
Averaged E/e’ ratio	8 ± 2.1
TAPSE (mm)	18.8 ± 4.7
RV FAC (%)	45 ± 7
coronary dilatation	6 (26)
coronary thrombosis	1 (4)
pericardial effusion	6 (26)
**CARDIAC MRI (*n* = 17/23)**	
LVEF	60 ± 13
LV edema	1 (4)
LV LGE	6 (26)
RVEF	62.8 ± 3.9

**Table 3:** Cardiac findings in patients with or without myocardial injury. Values are numbers (%) or mean ± standard deviation. Legend: BNP (brain natriuretic peptide); CPR (C-reactive protein); ERS (Erythrocyte Sedimentation Rate); GLS (global longitudinal strain); LVEF (left ventricular ejection fraction); LV LGE (left ventricle late gadolinium enhancement); MIS-C (multisystem inflammatory syndrome in children); PCT (procalcitonin); RV FAC (right ventricle fractional area change); RVEF (right ventricular ejection fraction); TAPSE (tricuspid annular plane systolic excursion); TnI (troponin I). * = *p* < 0.05.

	TnI+ (*n* = 15)	TnI− (*n* = 8)	*p*-Value
BNP (pc/mL)	824 ± 815	125 ± 105	0.03 *
CRP (mg/L)	205 ± 104	135 ± 49	0.038 *
PCT (ng/mL)	62.6 ± 118	31.1 ± 44.8	0.54
ESR (mm/h)	62 ± 36	49 ± 37	0.42
D-dimer (mcg/mL)	2032 ± 3112	2834 ± 2394	0.53
SARS-CoV-2 Ig title (kAU/L)	11.6 ± 28.1	10.3 ± 8.6	0.90
echo LVEF (%)	56 ± 10	65 ± 8	0.04 *
GLS (%)	−16.1 ± 4.2	−19.8 ± 3.2	0.04 *
E/A ratio	1.99 ± 0.51	1.9 ± 0.54	0.70
Averaged E/e’ ratio	8.2 ± 1.8	7.7 ± 2.6	0.64
TAPSE (mm)	20 ± 4.9	16.4 ± 3.4	0.09
RV FAC (%)	46 ± 8.2	44 ± 4.9	0.63
coronary dilatation	6 (40)	2 (25)	0.47 (X^2^ 0.52, *p* 0.65 FET)
pericardial effusion	3 (20)	3 (37)	0.36 (X^2^ 0.82)
**CMR (*n* = 17)**	**TnI+** **(*n* = 13)**	**TnI −** **(*n* = 4)**	***p*-Value**
CMR LVEF (%)	59.4 ± 11.4	64 ± 1.3	0.38
CMR LV LGE	4 (30)	2 (50)	0.91 (X^2^ 0.01, *p* 0.58 FET)

## Data Availability

The data presented in this study are available on request from the corresponding author.
